# Coronavirus disease 2019 in proportion to population: a historical analysis of Saudi Arabia

**DOI:** 10.1186/s42269-022-00876-z

**Published:** 2022-07-07

**Authors:** Asharaf Abdul Salam, Rshood M. Al-Khraif, Thandassery R. Dilip, Ibrahim Elsegaey

**Affiliations:** 1 King Saud University Center for Population Studies, PB No. 2454, Riyadh, 11451 Saudi Arabia; 2 International Institute for Population Sciences, Mumbai, India

**Keywords:** Social impact, Families, Major affected localities, Recoveries, Mortality

## Abstract

**Background:**

Saudi Arabia is one of the countries seriously affected by coronavirus disease 2019 (COVID-19) worldwide. With a few cases in early March, the daily spread of this disease increased to nearly 5000 at one point in time during the first wave to mid-June 2020. With committed efforts and public health interventions, it has been controlled to nearly 1000 by the end of August 2020 and less than 217 by November 28, 2020; thereafter, reporting declines and small increases. However, by December 2021, a third wave started, lasting for 2 months, during which the infection rate increased rapidly. By April 1, 2022, the number of infected persons in the country was 750,998, with 9047 deaths, 7131 active, and approximately 400 critical cases. This analysis of COVID-19 statistics of the Ministry of Health of Saudi Arabia (March 2020–April 2022) is carried out along with population data to extract patient proportions per 100,000 persons to illustrate the hypothesized social and community impact, which influences families and households.

**Results:**

The results showed a high rate of infection and mortality, but with recovery. These rates varied across localities and cities. A few cities with higher population densities are less affected by the spread of the epidemic. However, few localities and upcoming cities/townships were severely affected. These effects are explained as the percentage of the population affected, which exposes the impact on societies, families, and individual members. With concerted efforts, they are brought under control through recovery and adopting mitigation methods.

**Conclusions:**

Localities could be classified into four categories based on the proportion of the infected population: rapidly increasing, moderately increasing, declining, and stabilizing. Moreover, differential proportions of the affected population have implications at social and familial levels. Analysis and understanding of these trends, considering the base population, are important for policy building and intervention strategies accounting for grassroots-level demographics, which might serve as a tool to enhance interventions at population and family levels. Strategies for awareness creation and compassionate care are essential to address the psychosocial impact of health emergencies, as proved by the Ministry of Health, Saudi Arabia.

## Background

The outbreak of coronavirus disease 2019 (COVID-19), as stated by Zhou et al. (2020), created tensions even within well-knit family units, especially in poor infrastructure conditions and crowded living arrangements. Such tensions changed family and social life, restricting interpersonal contacts and affective gestures within the small circle of people, as well as strict disciplined precautions of social distancing, protection of face masks, and no handshakes (Iqbal et al. 2020). This led to containment, quarantine, lockdowns, and curfews (Zhou et al. 2020). Such isolations implemented to curb disease spread paved the way for conflicts, tensions, and violence: physical, psychological, and financial (Usher et al. 2020). In contrast, COVID-19 manifests differently among family members depending upon contact: some are infected but not others because of different immune responses (Sun et al. 2020).

As a result, there have been enormous changes and unparalleled consequences in the society and families, including financial, resulting in a demand for government support (Hitchings and Maclean 2020; Michels et al. 2020). Therefore, social distancing, isolation, quarantine, and lockdown strategies for epidemic transmission control management are central to the public health strategy toward mitigation and containment, which in turn influences social and family dynamics and economics (Usher et al. 2020; Humphreys et al. 2020; Motlhatlhedi et al. 2020).

In Saudi Arabia, the pandemic spread was addressed in the Riyadh declaration on digital health formulated in August 2020 to create the infrastructure necessary for effective evidence-based practices and high-quality real-time data aimed at providing actionable information to more health systems and countries (Al-Knawy et al. 2020). Although attempts to address COVID-19 spread have been assessed and investigated from a medical perspective in addition to the daily data release from the Ministry of Health, assessments of economic, social, and community impacts have not been carried out, especially at the social and family levels (Almaghlouth et al. 2020). However, there are few analyses indirectly exposing the family to this issue. For example, a study of oncology management experience during the epidemic explains the use of national and international guidelines to minimize infection risk and subsequent complications mentioning the risk of infection from healthcare staff to other family members (Ibrahim et al. 2020). Another study revealed moderate to severe psychological impact in one-fourth of the general population (Alkhamees et al. 2020), whereas another study explained the negative psychological effect of COVID-19 on physicians (Al-Sulais et al. 2020). Barry et al. (2020a) narrated the spread of Middle East respiratory syndrome coronavirus (MERS-CoV2; COVID-19) in the Kingdom. In addition, Barry et al. (2020b) and Salam et al. (2022) explained the case fatality rate, transmission exacerbating factors, and awareness of disease spread as a result of the Kingdom’s swift community action and hospital preparedness toward mitigation. All these studies have highlighted the importance of population, society, and family in addressing the disease. Still, many more investigations are necessary to succeed in such emergency situations, especially the demographic and public health analyses bringing out various aspects of population -, society, family, and individuals. More than small local-level survey-based research, national-level data analysis and macro-level interpretations are needed to enlighten policies and intervention strategies in the country. Here, we explain the national scenario by considering population size as denominator.

This consolidation was conducted to discuss the proportion of the population affected by COVID-19 and its probable influence on societal and family dynamics. This study has been carried out with the hypothesis that a large part of the statistics is misunderstood and misleading, without exposing the denominator, the population at risk, which shows the disease load and burden at the society, community, and family levels. There are countries with a population of a few million to a hundred million, or even a billion. The number of infected persons is treated as a percentage of the total population, which provides scope for understanding the number of infected persons per family. Although data availability at the national level by demographic, social, or economic characteristics raises concerns, this analysis is important.

## Methods

This analysis is based solely on the daily status reports of the Ministry of Health, Saudi Arabia, from its beginning on March 21, 2020 to April 1, 2022, giving the number of infections recorded at various localities. Calculations are performed with these data based on total population size, at the national level, accessed online (worldometers, populationstats, and worldpopulationreview), at various points during 2021 and 2022. Adopting population size (35,257,261 on April 28; 35,301,732 on May 28; 35,349,168 on June 28; 35,393,638 on July 28; 35,439,591 on August 28; 35,484,062 on September 28; 35,575,968 on November 28; 35,626,369 on December 28, 2021; 35,712,345 on February 28; and 35,753,851 on March 28, 2022, cited on www.worldometers.info) for each month, as the denominator, the following indicators are calculated for the country as a whole:

- Daily reported cases per 100,000 population
- Total cases and total recoveries per 100,000 population
- Total deaths per 100,000 population
- Critical cases per 100,000 population
- Active cases per 100,000 population

Calculations are performed to show the affected locations and the 100,000 population. In addition, they were presented in a graph and divided into four groups. Such analyses were performed to quantitatively explore the effect. Furthermore, with the available information on the number of cases in various localities and their corresponding population data gathered from the above-mentioned sources, their rates per 100,000 population were calculated, tabulated, and discussed. This is a proxy for the number of persons affected by the family. On the other hand, it explains the seriousness or threat to society. All these available statistics could lead to a situation analysis; the non-availability of further breakups limits the scope to a large extent. However, this preliminary analysis gives a lead, input, and indication for further analysis and extensions.

## Results

Results of analyses are given below under two heads.

### Epidemic infection as proportion of population

**Fig. 1:**
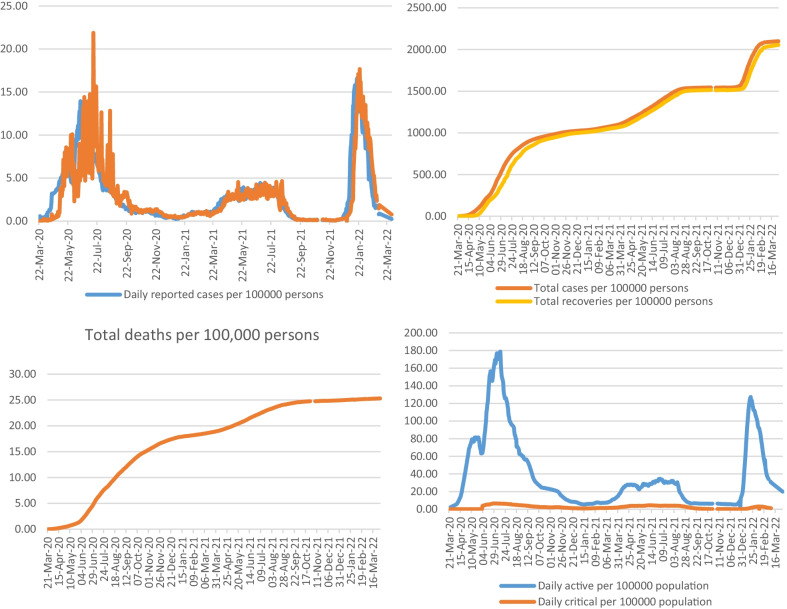
Spread of COVID-19, recoveries and fatality per 100,000 persons

There are a total of 750,998 infected cases, as of April 1, 2022 (2100 per 100,000 persons), with higher infection rates, but almost all recovered (734,820; 2055 per 100,000 persons), while the rest either passed away (9047; 25 per 100,000 persons) or were under treatment (7131; 20 per 100,000 persons). Thus, there is a narrow gap between infected and recovered persons, especially in the first two waves but different in the third wave. However, they are bridged at a faster pace, as shown in Fig. 1. It is the mortality from COVID-19 which has received greater concern in the country due to its ups and downs.

This higher cause-specific mortality creates a large burden on the public health system. However, daily reports of cases also show reductions in infections and mortality coupled with intermittent increases in infections and case fatality. With healthcare interventions, there were reductions in cases and mortality, showing firm commitments and accountability, which could be modeled to regain family solidarity in epidemic-prone situations, especially the healthy mix of awareness creation and compassionate care.

While applying the total population as the denominator, daily infection of COVID-19, as plotted, reached its peak point of 14 per 100,000 persons on June 17, 2020, corresponding to the highest number of infections of 4919 in the country, reported on a particular day. On the same day, the total number of cases was 401 per 100,000 people. With strenuous efforts and health interventions, these rates have steadily reduced. It is to be noted that the highest recovery of 7718 (22 per 100,000 persons) was recorded on July 14, 2020. However, the number of daily infections per 100,000 persons from its highest mark declined sharply to 1 on February 28, which increased to 3 on April 30, 2021. While the daily cases declined to none per 100,000 persons on January 3, 2021, and increased thereafter. Similarly, total cases declined in 2021 to 1030 on January 3, 1070 on February 28, and 1184 on April 30 per 100,000 persons; total recoveries were 1005, 1044, and 1136; and total deaths were 18, 18, and 20 per 100,000 persons, respectively. From December 2021–January 2022, the third wave and daily infections reached a height of 5928 on January 19 (17 per 100,000 persons with a recovery of 14 per 100,000 persons).

### Major localities including future cities

Saudi Arabia is divided into 13 administrative areas and 118 governorates. Reports of COVID-19 considered a mixed geographical pattern of 213 affected localities in the country. Certain localities with higher infection per household were identified from the data. The top 30 localities in each time period, including other future cities at various months, gave rise to a total of 48 localities. These localities vary in terms of population size; thus, this analysis across the number of affected persons per 100,000 persons was carried out using population size as of 2021, cited in the above-mentioned online sources (Table 1). Major localities, such as big cities, are seriously affected by the epidemic but are less intense when considering the density of infection on the specified parameters. For example, the largest city, the capital city of Riyadh, had the highest number of affected persons (58,589) until November 28, accounting for 1393 per 100,000 persons. Attention should be paid to neighborhoods such as Hofuf (6781), Dammam (2544), Dhahran (4373), Jazan (3255), Baish (2868), Bishah (1882), and Safwa (3616) for their higher number of affected persons per 100,000 persons. These are comparatively higher than those of other larger cities, such as Jeddah, Makkah, and Madinah.

**Table 1:** Infections per 100,000 persons in 30 major affected localities (during 2020)

No	Localities	31 Mar	30 Apr	31 May	30 Jun	31Jul	31Aug	30Sep	31 Oct	28 Nov
1	Riyadh^1a^	10	93	469	1092	1259	1306	1332	1356	1393
2	Jeddah^2a^	7	127	504	868	1044	1097	1152	1166	1186
3	Makkah^2a^	9	399	1146	1835	2155	2320	2447	2529	2584
4	Madina^3a^	6	291	731	1048	1267	1378	1474	1618	1705
5	Dammam^5a^	12	156	687	1576	2191	2339	2467	2514	2544
6	Hofuf^1^	4	355	1265	3691	5646	6092	6495	6697	6781
7	Qatif^5a^	57	170	914	5173	7448	7734	8187	8317	8383
8	Taif^2a^	28	309	1761	5458	1861	1983	2039	2062	2086
9	Al Jubail^5^	0	191	1132	1689	2024	2148	2254	2307	2332
10	Khobar^5^	15	151	1160	3153	4027	4182	4309	4355	4387
11	Najran^11a^	5	10	42	441	1148	1406	1524	1599	1648
12	Khamis Mushait^6^	2	12	49	699	1515	1755	1850	1947	2012
13	Bishah^6^	16	67	175	697	1451	1789	1854	1871	1882
14	Buraiyda^4a^	1	40	192	497	979	1206	1253	1308	1369
15	Baish^10^	–	341	952	1281	1847	2763	2859	2867	2868
16	Dhahran^5^	12	72	594	2203	3273	3617	4061	4248	4373
17	Abha^6a^	5	15	–	864	1876	2164	2279	2387	2424
18	Tabouk^7a^	1	49	160	290	566	758	803	812	843
19	Hail^8a^	–	11	122	532	1267	1721	1932	2088	2231
20	Hafr Albatin^5^	–	7	–	435	1181	1448	1508	1516	1523
21	Yanbu^3^	1	30	203	459	963	1345	1649	2103	2349
22	Jazan^10a^	7	63	–	–	1291	2684	3026	3209	3255
23	AlBaha^12a^	14	35	–	–	–	–	–	–	–
24	Unaiza^4^	–	34	49	201	530	718	757	811	875
25	Ad Diriya^1^	–	–	1083	2253	2346	2434	2442	2457	2462
26	Safwa^5^	–	–	462	2402	3329	3475	3586	3612	3616
27	Al Ahsa^5a^	0	–	–	–	–	–	–	–	–
28	ALZelfi^1^	–	111	194	–	–	–	–	–	–
29	AlMubarass^5^	–	–	–	882	2220	2442	2649	2766	2806
30	Wadi Addawasir^1^	–	–	–	657	1244	1526	1636	1790	1899
31	Saihat^5^	3	–	–	–	–	–	–	–	–
32	Raz Tanura^5^	1	14	90	376	–	–	–	–	–
33	Arar^9a^	1	18	–	–	–	–	–	–	–
34	Al Kharj^1^	–	–	50	215	368	410	426	446	456
35	Mahayel Asir^6^	–	–	–	251	608	628	670	692	701
36	Khulais^2^	–	381	1589	–	–	–	–	–	–
37	Bqeeq^5^	–	68	546	–	–	–	–	–	4974
38	Abu Arish^10^	–	–	–	–	–	2640	2939	2957	–
39	Al Hada	–	261	2237	–	–	–	–	–	–
40	Al Khafji^5^	4	–	–	–	–	–	–	–	–
41	Samtah^10^	4	–	–	–	–	–	–	–	–
42	Qunfudah^2^	0	–	51	–	–	–	–	–	–
43	Ar Raz^4^	1	–	–	–	–	–	–	–	–
44	Al Nairiya^5^	0	–	–	–	–	–	–	–	–
45	Al Dawadmi^1^	2	–	–	–	–	–	–	–	–
46	Al Majmaah^1^	–	–	594	–	–	–	–	–	–
47	Al Muzahmiah^1^	–	–	–	1254	–	–	–	–	–
48	Ahad Rufaidah^6^	–	–	–	–	963	–	–	–	–

Numbers in superscript refer to the administrative area of the neighborhood; ^1^Riyadh; ^2^Makkah Al-Mokarramah; ^3^Al-Madina Al-Monawarrah; ^4^Al-Qaseem; ^5^Eastern Region; ^6^Aseer; ^7^Tabouk; ^8^Hail; ^9^Northern Borders; ^10^Jazan; ^11^Najran; ^12^Al-Baha; ^13^Al-Jouf (administrative areas)

Figures represents cases per 1000 households; and cases per 100,000 person

^a^Future cities

**Fig. 2:**
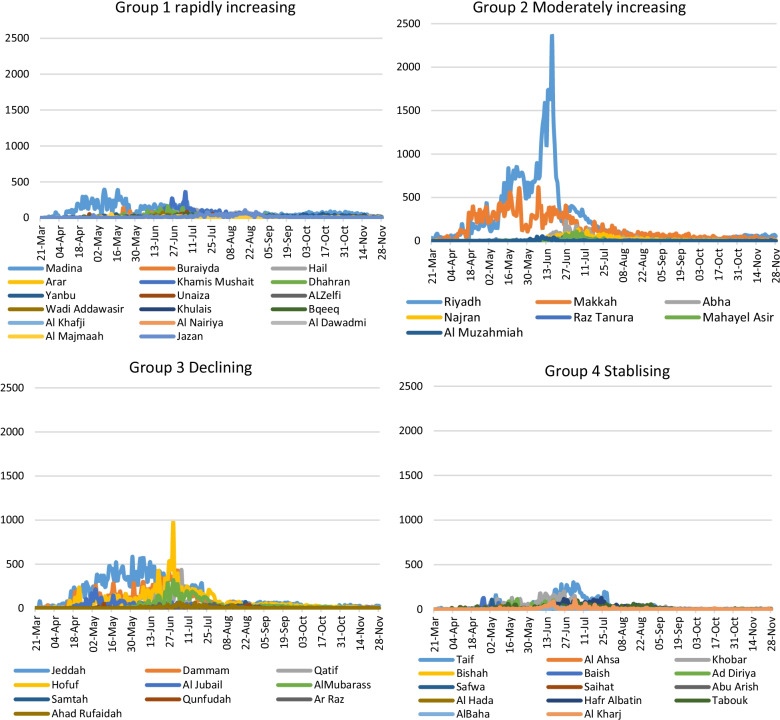
Neighborhoods classified according to the infection spread per 100,000 persons

This section has data available only up to November 28, 2020, where these localities are observed to fall under four categories based on the proportion of the affected population such as (a) rapidly increasing, (b) reasonably increasing, (c) slowly decreasing, and (d) stabilizing to a low level. The first group, which rapidly increases, includes five future cities, Madina, Buraydah, Hail, Arar, and Jazan, and 12 other localities, Khamis Mushait, Dhahran, Yanbu, Unaiza, AlZelfi, Wadi Ad Dawasir, Khulais, Bqeeq, Al-Khafji, Al-Nairiya, Al Dawadmi, and Al Majmaa. As a result, these localities rapidly increased on a daily basis (Fig. 2). The second group comprised of eight localities: five future cities of Riyadh, Makkah, Najran, Sakaka, and Abha, and three other neighborhoods of Raz Tanura, Mahayel Asir, and Al-Muzahmiah. The third category comprises ten localities: three future cities of Jeddah, Dammam, and Qatif, and seven other localities of Al Jubail, Al-Mubarass, Samta, Qunfudah, Ahad Al Rufaidah, Ar Raz, and Hofuf. The last category had 14 localities: four future cities of Taif, Al Ahsa, Tabouk, and Al Baha, and ten other localities of Bishah, Ad Diriyah, Saihat, Al Hada, Khobar, Baish, Hafar Al Batin, Safwa, Abu Arish, and Al Kharj.

Although these data have not been updated, it is assumed that the trend continued, keeping in mind the mass control programs executed in major administrative areas, locations, and cities. In addition, overcrowded and densely populated urban pockets are equipped with resources of manpower and materials to address epidemics than the less populated, rapidly developing urban pockets. Such trends have implications for individuals, families, communities, and societies in controlling infections, particularly in protecting people from the threat of epidemics.

## Discussions

The daily reported cases in the country increased since its inception in early March 2020, reaching its peak in July 2020 and declining thereafter, but with a less threatening second wave and a more threatening third wave. As pointed out by Stokes and Patterson (2020), population size and density are potential sources of COVID-19, especially in Arab countries. In such settings, disease prevention raises serious concerns because of the potential impact of lockdowns, isolation, and quarantine (Fisher et al. 2020; Luttik et al. 2020; Joshi et al. 2020; Patnaik and Maji 2020; Brock and Laifer 2020). The resultant disturbances lead to mental health issues associated with anxiety, depression, insomnia, stress, and obsessions in various sections of the population, including children, older adults, and vulnerable sections (Joshi et al. 2020). As applied to Saudi Arabia, especially in the major cities characterized by modern lifestyles under nuclear families, this situation affected traditional family togetherness and cohesiveness. Nevertheless, the gap between reported and recovered cases has been reduced to a minimum, enabling successful treatment outcomes, albeit with few departures (Salam et al. 2022).

There were increases in the overall total cases and mortality, which were attributed to the waves of this epidemic on a global basis. However, threats are limited, as revealed by affected persons as a percentage of the population. Still, the epidemic outbreak in the country arouses curiosity about the changing social and cultural values of people and families at various localities across the country, creating threats. Thus, population rates are more meaningful than absolute numbers for understanding their impact on societies and neighborhoods. These rates indicate the extent to which COVID-19 has impacted the population: distribution, economy, behavior, and cohesiveness, both directly and indirectly. As of June 17, 2020, the daily reported cases reached a figure of 14 per 100,000 persons, fluctuating afterward with significant declines. Later, during the third wave, it hits 17 on January 19, 2022. Such increases threaten societies, families, and individuals, especially when the density of infected individuals is higher. It has many consequences on social, economic, and psychological health and manifests in many forms of distress.

Recoveries from COVID-19 rose faster by November 28, 2020, and thereafter, the gap between total cases and recoveries appeared to be converging, giving hope for no gap very soon, both at the family and individual levels a symbol of achievements of the mitigation strategy adopted. It will relieve society and family from many disturbances and suffering, causing burdens in the long run. At this point, efforts are made to achieve maximum recovery while minimizing infections. However, there was no decline in the number of deaths due to COVID-19. This could be due to many factors, such as the age of the infected person, population age structure, and comorbidities. Unfortunately, data on such demographics are unavailable for computation. None of the countries started offering data on the age and sex of patients with COVID-19 at the national level. Small sample surveys conducted at certain locations have generated such data but are insufficient for national-level interpretations.

However, large urban pockets have a smaller proportion of families and persons affected than certain medium-sized urban pockets. This raises concerns and considerations of increasing coverage of health intervention as well as familial support to medium-sized urban localities such as Hafar Al-Batin, Hofuf, Bisha, and Baish. These upcoming towns recorded a rapid spread and were analyzed as a share of the population. Such instances have serious repercussions on people’s lives at various levels of family and society, including the burden of epidemics, anxiety, and related neurotic conditions apart from the economics.

Manifestations to those in contact depend upon immunity leading to family tragedies and long progression causing widespread and interconnected human suffering: deaths, critical illness, disrupted economies, and social structures apart from the indirect effects of intense external boundaries of physical and emotional contacts, virtual connections, and migrations (Sun et al. 2020; Lebow 2020a, b; Luttik et al. 2020; Humphreys et al. 2020; Fraenkel and Cho 2020). Such ill effects could be curbed through interventions, especially awareness creation, combined with support systems. The government of Saudi Arabia offers several economic and medical welfare measures to protect the affected families (Ministry of Media 2020). These interventions should be continued for a further period, at least until the epidemic disappears, which is until the recovery of all patients, as advocated by Stokes and Patterson (2020) and Salam et al. (2022).

A rapidly increasing epidemic progression per population has been observed in most localities, especially in medium-sized cities and towns, rather than in metropolitan cities with a large population, including migrants. The progression in Qatif is the highest, followed by Hofuf, Khobar, and Dhahran in the order while analyzed as per 100,000 population. These northern localities border Kuwait, Bahrain, and Qatar; thus, frequent international traveling which appears to be the widest spread neighborhood, followed by Bisha and Hofuf. The progression of epidemics in these localities deserves serious consideration. This clearly explains the increased risk of infection, pointing out the possible flaws in the implementation of precautionary measures as per the recommendations. Large cities confined to procedural directions could reportedly control the spread to a large extent with existing infrastructural and social networking. However, the five large cities of Riyadh, Jeddah, Makkah, Madina, and Dammam have experienced a slow progression of COVID-19 in comparison with the upcoming townships. Thus, with this lesson in front, the Ministry of Health may take immediate steps for the strict implementation of control measures in these localities.

The results of this analysis show that the extent of the country’s population is affected, as are characteristics such as population size, density, and infrastructural development influencing spread of COVID-19. While the major cities with high population pressure could cope with the rapid spread of the epidemic in a short span, the upcoming medium-sized cities took a bit more time. It is a learning that such urban townships demand more care, concern, and control during instances of epidemics and similar calamities. Therefore, it can be inferred that a certain level of infrastructure networking is essential to address calamities, including epidemics, for which larger cities have an advantage.

Analyses and interpretations accounting for the total population as the denominator reflect the possible impact on the population and thereby society and family, which serve as a tool to address life at the grassroots level with welfare and benefits to uplift those in distress. This analysis was carried out with minimum publicly available data. However, this could enlighten academics to the extent possible. Detailed analyses are not amenable with this dataset, which is a limitation of this study. It would be advisable to analyze data at the national level for age, sex, and socioeconomic status through interventions that could teach lessons for the future. Such analyses are possible with the actual database of the Ministry of Health but not with the published capsuled data. We hope the researchers get an opportunity in the future to access the database and bring out detailed research reports and manuscripts for the benefit of the larger society.

## Conclusions

The proportion of the population infected in society deserves special attention during an epidemic for many reasons, as it influences personal relations, intimacy, financial conditions, common forms of interpersonal communication, and patient care mechanisms. Day by day, the disease burden increases with the number of positive cases and fatalities but is comforted with recovery and improvement of living conditions. Although major cities in Saudi Arabia are negatively affected, they are less affected by population size than medium-sized upcoming cities and townships. Nevertheless, the probability of a rapid spread in densely populated metropolitan pockets and future cities cannot be neglected. Therefore, future-oriented welfare measures and policies to uplift populations and societies in crisis are required to save them from socioeconomic and relationship-related crises associated with the physical threats of COVID-19.

## Data Availability

This research used a compilation of daily status reports published by Ministry of Health, Saudi Arabia (www.moh.gov.sa). The compiled data, in Excel worksheet, may be made available on request.
